# The Plasma Proteome Is Associated with Anthropometric Status of Undernourished Nepalese School-Aged Children^1^,^2^,^3^

**DOI:** 10.3945/jn.116.243014

**Published:** 2017-02-01

**Authors:** Sun Eun Lee, Christine P Stewart, Kerry J Schulze, Robert N Cole, Lee S-F Wu, James D Yager, John D Groopman, Subarna K Khatry, Ramesh Kant Adhikari, Parul Christian, Keith P West

**Affiliations:** 4 Center for Human Nutrition, Department of International Health, and; 5 Department of Environmental Health and Engineering, Johns Hopkins Bloomberg School of Public Health, Baltimore, MD;; 6 Program in International and Community Nutrition, Department of Nutrition, University of California Davis, Davis, CA;; 7 Mass Spectrometry and Proteomics Facility, Department of Biological Chemistry, Johns Hopkins School of Medicine, Baltimore, MD;; 8 Nepal Nutrition Intervention Project-Sarlahi, Kathmandu, Nepal; and; 9 Kathmandu Medical Collage, Kathmandu, Nepal

**Keywords:** plasma proteome, anthropometry, mass spectrometry, children, Nepal, insulin-like growth factor I, insulin-like growth factor–binding proteins

## Abstract

**Background:** Malnutrition affects body growth, size, and composition of children. Yet, few functional biomarkers are known to be associated with childhood morphology.

**Objective:** This cross-sectional study examined associations of anthropometric indicators of height, musculature, and fat mass with plasma proteins by using proteomics in a population cohort of school-aged Nepalese children.

**Methods:** Height, weight, midupper arm circumference (MUAC), triceps and subscapular skinfolds, upper arm muscle area (AMA), and arm fat area (AFA) were assessed in 500 children 6–8 y of age. Height-for-age *z* scores (HAZs), weight-for-age *z* scores (WAZs), and body mass index–for-age *z* scores (BAZs) were derived from the WHO growth reference. Relative protein abundance was quantified by using tandem mass spectrometry. Protein-anthropometry associations were evaluated by linear mixed-effects models and identified as having a false discovery rate (*q*) <5%.

**Results:** Among 982 proteins, 1, 10, 14, and 17 proteins were associated with BAZ, HAZ, MUAC, and AMA, respectively (*q* < 0.05). Insulin-like growth factor (IGF)-I, 2 IGF-binding proteins, and carnosinase-1 were associated with both HAZ and AMA. Proteins involved in nutrient transport, activation of innate immunity, and bone mineralization were associated with HAZ. Several extracellular matrix proteins were positively associated with AMA alone. The proteomes of MUAC and AMA substantially overlapped, whereas no proteins were associated with AFA or triceps and subscapular skinfolds. Myosin light-chain kinase, possibly reflecting leakage from muscle, was inversely associated with BAZ. The proteome of WAZ was the largest (*n* = 33) and most comprehensive, including proteins involved in neural development and oxidative stress response, among others.

**Conclusions:** Plasma proteomics confirmed known biomarkers of childhood growth and revealed novel proteins associated with lean mass in chronically undernourished children. Identified proteins may serve as candidates for assessing growth and nutritional status of children in similar undernourished settings. The antenatal micronutrient supplementation trial yielding the study cohort of children was registered at clinicaltrials.gov as NCT00115271.

## Introduction

Child growth restriction is the result of complex metabolic adjustments to acute or prolonged malnutrition (1). However, our understanding of the biological pathways underlying decelerated linear growth and reduced lean and fat mass remains inadequate. Although anthropometry is the conventional method for assessing growth because of its simplicity, quantitative nature, interpretability, widespread use, and low cost, it offers little biological insight. It may not be sensitive to changes in many aspects of nutritional status and fails to discern complex causes of growth deficit (2). We suggest that novel, valid, and reliable panels of biomarkers can be discovered by exploiting omics capabilities. These molecules may reflect nutritionally regulated biological pathways affecting growth, body composition, function, and development that may be suitable for public health application.

Plasma proteomics is the study of detecting, quantifying, and analyzing proteins present in the plasma (3). It may offer the potential to reveal biological pathways and discover novel protein markers of childhood growth and body composition. The unbiased (i.e., hypothesis-generating) approach of plasma proteomics can allow comprehensive and simultaneous analysis of many circulating and some tissue-leaked proteins reflecting anabolic or catabolic tissue metabolism (3). For example, the growth hormone–insulin-like growth factor (IGF)^10^-I axis has long been considered the main regulatory system of childhood growth (4). It has endocrine, autocrine, and paracrine growth-promoting effects in most tissues in the body and shows high sensitivity and specificity to indicators of nutritional status (5). However, many other growth factors and intracellular and extracellular matrix proteins are also critical to regulate cellular activity, providing structural and biochemical support for local tissue growth (6, 7). In addition to hormonal and local control, growth and body composition of growing children living in impoverished areas are likely to be affected by a complex interplay between nutrient metabolism and the immune and neurologic systems (8). Thus, the global discovery mode of plasma proteomics may help to advance our understanding of dynamic structural or regulatory mechanisms of tissue growth associated with nutritional or other environmental exposures.

We have assessed nutritional, health, and cognitive status of a population-based birth cohort of school-aged children in the rural plains of Nepal (9–12). In this region, growth restriction starts in fetal life (13) and persists through the preschool- and school-aged years, manifested by low *z* score distributions in height-for-age (HAZ), BMI-for-age (BAZ), and weight-for-age (WAZ) (10). As a substudy, we have applied quantitative proteomics to reveal plausible biological associations between circulating plasma protein abundance and the nutritional and health status of children. We have demonstrated predictive biomarkers of current micronutrient status and inflammation (14–17) and have identified proteins prospectively associated with cognitive function (18). The strength, stability and broad nature of associations suggest that the plasma proteome may have the potential to identify functional biomarkers of depressed childhood linear and ponderal growth as well as alterations in body composition. Therefore, the proteome may provide unique opportunities to reveal underlying biological mechanisms of malnutrition and poor growth.

In this study, we hypothesized that proteins associated with attained skeletal bone length, musculature, and subcutaneous fat deposition assessed by anthropometric measures and that indicators exist and can be detected and quantified in plasma via an untargeted proteomics approach. Our findings may offer novel insights into pathways and networks that affect growth and body composition and reveal biological links to risk factors and health consequences of child undernutrition in a rural South Asian setting.

## Methods

### 

#### Study population and design.

In 1999–2001, a population-based, randomized controlled trial of antenatal micronutrient supplementation was carried out to improve birth outcomes and infant survival in the Sarlahi District, a rural southeastern region of Nepal (NCT00115271) (13, 19). In 2006–2008, the surviving children born during the trial were followed up to examine the effects of the maternal intervention on growth, body composition, and metabolic health. Details of the child follow-up study and results have been previously reported (10, 11). Children in the present study comprised a stratified, random subset of the trial cohort (**Supplemental Figure 1**) (14). Among 4130 live-born infants during the maternal micronutrient supplementation trial, 3524 children were followed up at 6–8 y of age. Of 3305 children with available blood samples, 2130 (64%) met imposed inclusion requirements for this analysis of having adequate plasma sample volume and complete epidemiologic data from both the original trial and follow-up study to permit full exploration of admitted child records. We stratified data from eligible children by the original 5 maternal supplement allocation groups and ordered them by calendar date of blood draw in the field during the follow-up study. Two hundred children per supplementation group were systematically selected for micronutrient assessment (*n* = 1000). Their comparability to the larger group of children with respect to distributions of nutritional and health variables has been described (9). Of these, 50% (*n* = 500 samples), balanced across maternal intervention groups (*n* = 100 each) were randomly selected for proteomics analysis. All research activities were restricted to children whose parents provided informed consent. Institutional Review Boards at the Institute of Medicine of Tribhuvan University, Kathmandu, Nepal, and at Johns Hopkins University, Baltimore, Maryland, reviewed and approved the research protocols of both the original trial and follow-up study.

#### Field data collection and anthropometric measurements.

Standardized, home-based anthropometric measurements obtained on children have been described elsewhere (10). Briefly, standing height (in centimeters) was measured by using Harpenden portable stadiometers (Harpenden), weight (in kilograms) was measured with children lightly clothed by using an electronic scale (Model 881; Seca), and midupper arm circumference (MUAC) (in centimeters) was measured at the midpoint between the acromion and olecranon processes of the left upper arm with an insertion tape (20). Triceps skin fold (in millimeters) was measured at the midpoint of a back upper arm, and subscapular skin fold (in millimeters) was measured ∼2 inches (5.08 cm) below the lateral angle of the shoulder blade with Holtain precision calipers (Holtain Ltd). All measurements were repeated 3 times, and median values were used for analysis. HAZs, BAZs, and WAZs were calculated based on the WHO growth reference for children and young adults 5–19 y of age (21). Arm muscle area (AMA; in square centimeters) and arm fat area (AFA; in square centimeters) were calculated by using equations described by Stewart et al. (10). During another home visit near the time of anthropometric assessment, children were asked to fast overnight after which trained phlebotomists drew early-morning blood samples (11). At a field laboratory center, the samples were centrifuged, and plasma was extracted, aliquoted, and stored at −20°C in freezers. The frozen samples were shipped in liquid-nitrogen dry-vapor shippers to the Center for Human Nutrition at the Johns Hopkins Bloomberg School of Public Health and stored at −80°C.

#### Proteomics analysis.

Details of quantitative proteomics processes have been reported elsewhere (14). In brief, 6 high-abundance proteins (albumin, IgG, IgA, transferrin, haptoglobin, and antitrypsin) were immune depleted from the plasma samples of children (40 μL) by using a Human-6 Multiple Affinity Removal System LC column to enhance detection of low-abundance proteins. At the MS and Proteomics Core in the Johns Hopkins School of Medicine samples were digested with trypsin overnight. Seven samples and 1 pooled sample were labeled with isobaric Tags for Relative and Absolute Quantitation (iTRAQ) 8-plex reagents. The pool of all 8 samples was fractionated into 24 fractions by strong cation exchange chromatography and loaded to a reverse-phase nanobore column. Eluted peptides were sprayed into an LTQ orbitrap Velos mass spectrometer interfaced with a NanoAcquity ultra-HPLC. Precursor and the fragment ions were analyzed, and MS/MS spectra were extracted and searched against the RefSeq 40-protein database by using Mascot through Proteome Discoverer Software. Peptides with ≥95% confidence were filtered for peptide identification. A total of 72 iTRAQ experiments were carried out for 500 samples in this study.

#### Statistical analysis.

Distributions of anthropometric measurements and *z* score indexes were checked for normality, and extreme values (<1%) were excluded from the analysis. Procedures for estimating relative abundance of proteins from reporter ion intensities across all MS spectra have been published elsewhere (22). Linear mixed-effects (LME) models were used to take into account the variability in proteomics data from multiple iTRAQ experiments. Random-intercept models were fitted with each anthropometric measurement or index as a dependent variable, proteins as fixed variables, and each iTRAQ experiment as a random effect. We did not adjust for maternal micronutrient supplementation because it did not significantly affect either plasma protein or anthropometric profiles in this small subset of children (SE Lee, unpublished results, 2015). Parameters were estimated by using restricted maximum likelihood estimation (23). *P* values were calculated by using a 2-sided test of a null hypothesis that there is no association between individual proteins and an anthropometric outcome. Multiple-hypothesis testing was corrected by controlling the false discovery rate (24). Proteins passing a false discovery rate threshold of 5% (*q* < 0.05) were considered significant. *R*^2^ was estimated by squaring a correlation coefficient (*r*) between an outcome variable and its respective best linear unbiased prediction from the LME models (25). In addition to proteins linearly associated with each anthropometric outcome, we identified proteins differentially abundant by dichotomized undernutrition status by fitting LME models with relative abundance of proteins as dependent variables, each undernutrition indicator as a fixed variable, and each iTRAQ experiment as a random variable.

Because data for proteins related to height-for-age were most complete, we generated a heatmap to illustrate each child’s LME model-predicted relative abundance for multiple proteins associated with the outcome. Because there were missing values for proteins of interest, we imputed values using multiple imputation 10 times (26) using predicted values from regression models between proteins and the outcome variable. We computed a row dendrogram from hierarchical clustering using a Pearson correlation method to calculate pairwise distances between proteins.

Because coregulated proteins in shared biological pathways can be expected to be correlated with each other, we have constructed a correlation matrix of all identified proteins in this study. We separately present a correlation matrix of proteins associated with AMA to illustrate potential biological clusters specific to arm musculature. We calculated Pearson correlation coefficients of pairwise protein-protein values in each iTRAQ experiment, and averaged correlation coefficients across all iTRAQ experiments were used. The order of proteins in the correlation matrix was determined by optimal leaf ordering that reorganizes proteins that are more correlated to be adjacent. The datasets of anthropometric measurements and indexes and relative abundance of plasma proteins used for analyses are available in **Supplemental Table 1**. All statistical analyses were performed by using the R Environment for Statistical Computing (version 3.1.2; R Development Core Team).

#### Protein annotation.

Human Genome Organization gene symbols of proteins were used in tables and text as abbreviations of protein names (27). Functional annotation clustering of proteins associated with anthropometric measurements was carried out to list common biological process terms through the Database for Annotation, Visualization and Integrated Discovery (DAVID, v6.7) by using Biological Process ontology of the Gene Ontology database (28–30). Additional information about molecular and biological functions of proteins was derived from the National Center for Biotechnology Information Protein Database and in-depth literature review (31).

## Results

**FIGURE 1:**
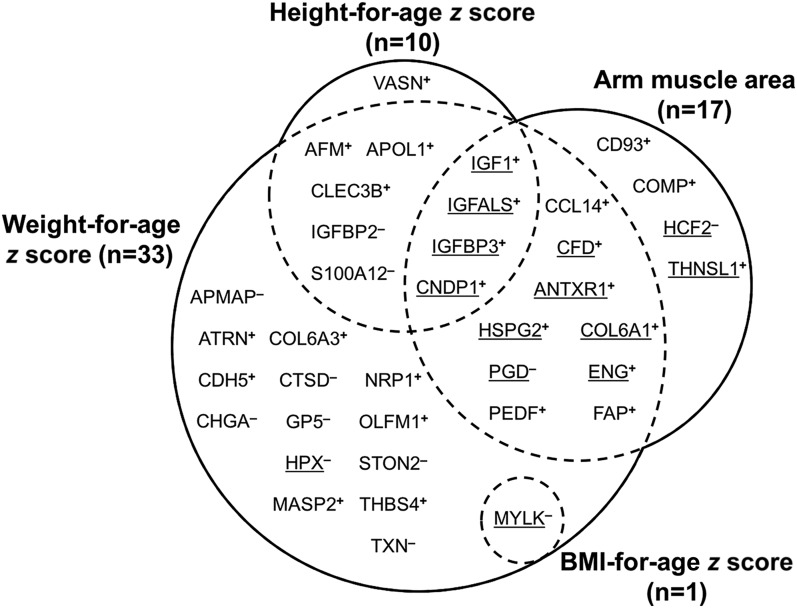
Venn diagram of the number of plasma proteins associated with height-for-age, BMI-for-age, and weight-for-age *z* scores and arm muscle area in 6- to 8-y-old Nepalese children (*q* < 0.05). Note: Proteins associated with midupper arm circumference (*n* = 14), comprising a subset of those associated with arm muscle area, are underlined rather than shown separately. Plasma proteins are indicated as their Human Genome Organization gene symbols. Superscript plus (^+^) and minus (^−^) signs indicate positive and negative associations, respectively. AFM, afamin; ANTXR1, anthrax toxin receptor 1; APMAP, adipocyte plasma membrane–associated protein; APOL1, apolipoprotein L1; ATRN, attractin; CCL14, chemokine (C-C motif) ligand 14; CDH5, cadherin 5; CD93, CD93 antigen; CFD, complement factor D; CHGA, chromogranin A; CLEC3B, tetranectin; CNDP1, carnosinase 1; COL6A1, collagen VI, α1; COL6A3, collagen VI, α3; COMP, cartilage oligomeric matrix protein; CTSD, cathepsin D; ENG, endoglin; FAP, fibroblast activation protein; GP5, platelet glycoprotein V; HCF2, heparin cofactor II; HPX, hemopexin; HSPG2, perlecan; IGFALS, insulin-like growth factor, acid labile subunit; IGFBP, insulin like-growth factor–binding protein; IGF1, insulin-like growth factor I; MASP2, mannan-binding lectin serine protease 2; MYLK, myosin light-chain kinase; NRP1, neuropilin 1; OLFM, noelin; PEDF, pigment epithelium-derived factor; PGD, phosphogluconate dehydrogenase; STON2, stonin-2; S100A12, protein S100-A12; THBS4, thrombospondin 4; THNSL1, threonine synthase–like 1; TXN, thioredoxin; VASN, vasorin.

Demographic characteristics and anthropometric measurements of the children in this study are presented in **Supplemental Table 2**. Children were undernourished as indicated by mean HAZs, BAZs, and WAZs of −1.79, −1.18, and −1.98, respectively, and mean MUAC, triceps skin fold, subscapular skin fold, AMA, and AFA values of 15.4 cm, 5.8 mm, 4.8 mm, 14.8 cm^2^, and 4.3 cm^2^, respectively. These are equivalent to mean values of <10th percentile for MUAC, triceps skin fold, and AFA, and <25th percentile for AMA and subscapular skin fold of distributions among well-nourished reference child populations in the United States (32–34). Among 982 proteins that were detected and quantified by MS in >10% of the all plasma samples (*n* > 50), 10, 14, 17, 1, and 33 plasma proteins were associated with HAZ, MUAC, AMA, BAZ, and WAZ, respectively (*q* < 0.05) (**Figure 1**). Four proteins were jointly associated with HAZ and AMA whereas most proteins associated with HAZ, BMZ, and AMA were also associated with WAZ. Except for 2, all proteins associated with MUAC were also associated with AMA. No proteins associated with indicators of fatness (AFA, triceps skin fold, and subscapular skin fold) passed the false discovery rate threshold of 5% (all *q* ≥ 0.05). Common Gene Ontology terms (categorized as Biological Process) for all identified proteins primarily included regulation of cell proliferation and growth, cell movement, skeletal system and muscle organ development, and inflammatory, defense, and wound healing responses (**Supplemental Table 3**).

### Plasma proteins associated with HAZ or stunting status.

Height-for-age was 0.17–0.80 of a *z* score higher per 50% increase in relative abundance of positively correlated proteins (**Table 1**). IGF-I, a growth-promoting factor (36), and 2 IGF-binding proteins [IGF-binding protein, acid labile subunit (IGFALS) and IGF-binding protein 3 (IGFBP3)] were positively associated with height-for-age, although the association was stronger with the binding proteins than IGF-I. Other positive correlates with HAZ included afamin and apo L1, nutrient transport proteins for vitamin E and lipids, respectively (37, 38); carnosinase 1, a carnosine degrading enzyme (39); and tetranectin and vasorin, involved in skeletal system and vascular development, respectively (40, 41).

**TABLE 1:** Plasma proteins positively and negatively associated with HAZ in 6- to 8-y-old Nepalese children (*q* < 0.05)^1^

Protein (gene symbol)	*n*^2^	Change in HAZ^3^	*R*^2^ ^4^	*q*^5^	Accession^6^	Molecular function or biological process^7^
Positive associations						
IGF-binding protein, acid labile subunit (*IGFALS*)	498	0.80 (0.65, 0.95)	0.30	1.91 × 10^−23^	4826772	IGF binding
IGF-binding protein 3 (*IGFBP3*)	498	0.59 (0.45, 0.74)	0.23	4.13 × 10^−13^	62243068	IGF binding
Afamin (*AFM*)	498	0.42 (0.22, 0.62)	0.15	0.0082	4501987	Vitamin E transport
IGF I (*IGF-I*)	172	0.31 (0.15, 0.46)	0.09	0.0128	163659901	Growth factor
Tetranectin (*CLEC3B*)	498	0.55 (0.26, 0.83)	0.14	0.0218	156627579	Bone matrix
Apolipoprotein L1 (*APOL1*)	498	0.32 (0.15, 0.5)	0.14	0.0282	211938442	Lipid transport
Carnosinase 1 (*CNDP1*)	498	0.17 (0.08, 0.27)	0.14	0.0401	21071039	Carnosine hydrolase
Vasorin (*VASN*)	498	0.39 (0.17, 0.62)	0.14	0.0495	88702793	Vasculogenesis
Negative associations						
Protein S100-A12 (*S100A12*)	375	−0.23 (−0.34, −0.13)	0.10	0.0045	5032059	Immune response
IGF-binding protein 2 (*IGFBP2*)	498	−0.28 (−0.44, −0.12)	0.14	0.0495	55925576	IGF binding

1 Ten proteins quantified by MS and estimated by linear mixed-effects modeling in >10% of the samples that were positively and negatively associated with HAZ (*q* < 0.05), listed by the direction and strength of association (in increasing order of *q*). HAZ, height-for-age *z* score; IGF, insulin-like growth factor.

2 Number of child plasma samples in which each protein was detected and quantified by MS. HAZ outliers (*n* = 2) were excluded; thus, the maximum number was 498.

3 Estimated change in HAZ (95% CI) of children per 50% (1.5 times) increase in the relative abundance of a protein.

4 Proportion of variability in HAZ explained by protein.

5 Multiple hypothesis testing was corrected by using the false discovery rate.

6 GenInfo sequence number as assigned to all protein sequences by the National Center for Biotechnology Information at the National Library of Medicine, NIH (35).

7 Represented or known molecular function or biological process of protein.

**FIGURE 2:**
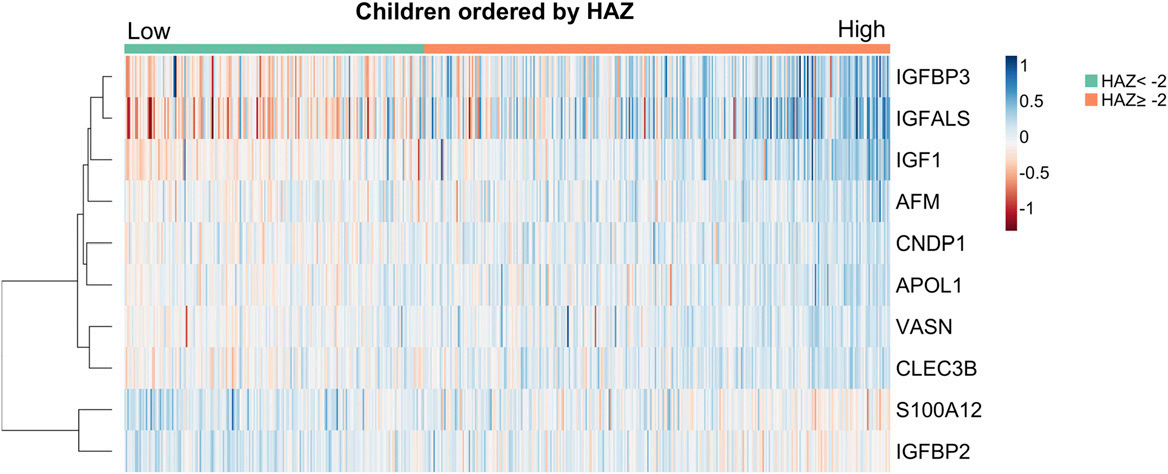
Heatmap of plasma proteins associated with HAZ in 6- to 8-y-old Nepalese children (*q* < 0.05). Each row and column denotes protein and individual child, respectively. Children (*n* = 498) were ordered by their HAZ in increasing order from the left to right side. The color of each cell indicates linear mixed-effects model–estimated relative abundance of protein. Blue and red indicate high and low abundance relative to the mean value of each protein, respectively. Row dendrogram (on the left side of heatmap) shows a hierarchical clustering of proteins based on their correlations. The shorter horizontal subbranches represent the higher correlations. AFM, afamin; APOL1, apolipoprotein L1; CLEC3B, tetranectin; CNDP1, carnosinase 1; HAZ, height-for-age *z* score; IGFALS, insulin-like growth factor, acid labile subunit; IGFBP, insulin-like growth factor–binding protein; IGF1, insulin-like growth factor I; S100A12, protein S100-A12; VASN, vasorin.

Height-for-age was lower by 0.23–0.28 of a *z* score per 50% increase in relative abundance of protein S100-A12 (S100A12), a protein involved in the innate immune response (42), and IGF-binding protein 2 (Table 1). In **Figure 2**, a heatmap displays increases or decreases in modeled relative abundance estimates for the 10 proteins positively and negatively associated with HAZ in increasing order (from the left to right side) among all analyzed children. This illustrates attained height-dependent variation in the plasma protein abundances. In a separate analysis, we also identified plasma proteins differentially abundant by stunting status. Five proteins including IGFALS, IGFBP3, carnosinase 1, and tetranectin, were 4–13% less abundant in the plasma of stunted children than nonstunted children (**Supplemental Table 4**).

### Plasma proteins associated with AMA.

**FIGURE 3:**
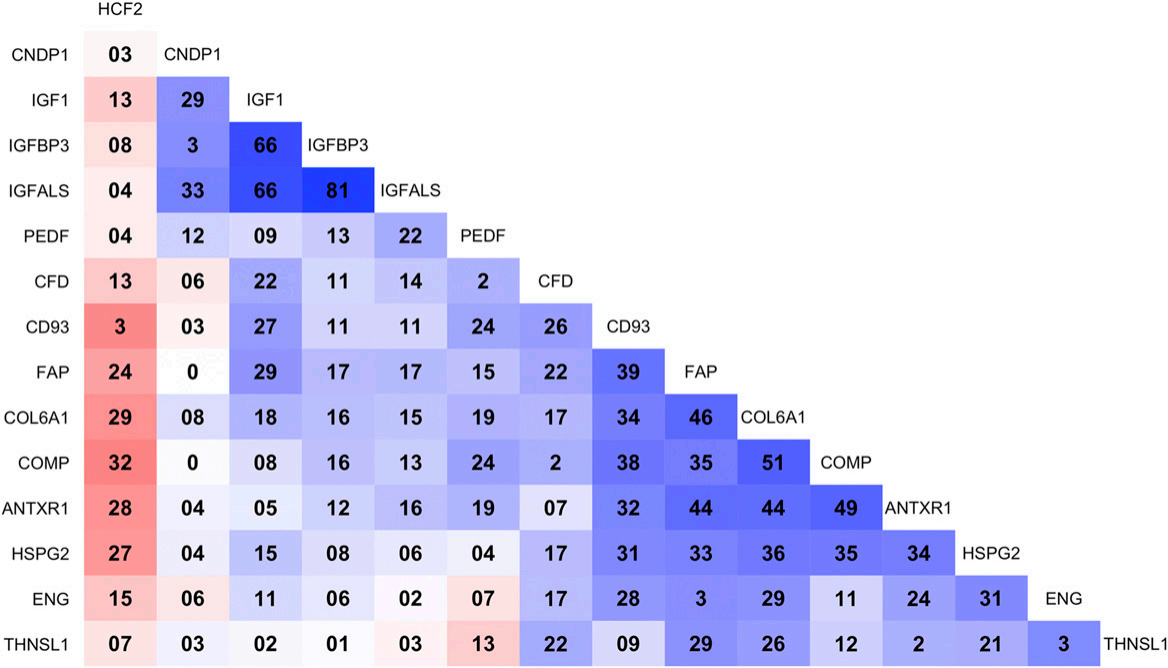
Matrix of correlation coefficients among plasma proteins associated with arm muscle area in 6- to 8-y-old Nepalese children (*q* < 0.05). Blue and red indicate positive and negative correlations, respectively, and darker colors represent a stronger association. All correlation coefficients (*r*) are presented as *r* × 10^2^ to improve visualization. Proteins with sample size <100 (*n* = 2) were not included. ANTXR1, anthrax toxin receptor 1; CD93, CD93 antigen; CFD, complement factor D; CNDP1, carnosinase 1; COL6A1, collagen VI, α1; COMP, cartilage oligomeric matrix protein; ENG, endoglin; FAP, fibroblast activation protein; HCF2, heparin cofactor II; HSPG2, perlecan; IGFALS, insulin-like growth factor, acid labile subunit; IGFBP3, insulin like-growth factor–binding protein 3; IGF1, insulin-like growth factor I; PEDF, pigment epithelium-derived factor; THNSL1, threonine synthase–like 1.

Fifteen and 2 proteins, respectively, were positively and negatively associated with AMA (**Table 2**). AMA was higher by 0.4–1.7 cm^2^ and lower by ∼1.0 cm^2^ per 50% increase in relative abundance of positively and negatively correlated proteins, respectively. Although relatively large in number (*n* = 17), most proteins explained only small percentages of its variation (*R*^2^ = 2–6%). We present a correlation matrix to reveal functional clusters of proteins that covary and likely interact within the plasma proteome of AMA in **Figure 3**. In this matrix we found high positive correlations between the IGF-I, IGFALS, and IGFBP3 proteins reflecting IGF-I ternary complex (*r* = 0.66–0.81) (43) and moderately high positive correlations (mostly *r* = 0.24–0.51) among another group of proteins. These include structural components of extracellular matrix, including collagen VI α1, cartilage oligomeric matrix protein, and perlecan (44), and receptors and molecules that interact to regulate extracellular matrix homeostasis, including CD93 molecule, fibroblast activation protein, anthrax toxin receptor 1, and endoglin (45–48).

**TABLE 2:** Plasma proteins associated with AMA in 6- to 8-y-old Nepalese children (*q* < 0.05)^1^

Protein (gene symbol)	*n*^2^	Change in AMA^3^	*R*^2^ 4	*q*^5^	Accession^6^	Molecular function or biological process^7^
Positive associations						
IGF-binding protein, acid labile subunit (*IGFALS*)	499	1.03 (0.64, 1.42)	0.05	1.00 × 10^−4^	4826772	IGF binding
IGF-binding protein 3 (*IGFBP3*)	499	0.82 (0.45, 1.18)	0.04	0.0043	62243068	IGF binding
CD93 antigen (*CD93*)	415	0.86 (0.42, 1.3)	0.03	0.0238	88758613	Cell adhesion
Collagen VI, α1 (*COL6A1*)	471	0.89 (0.43, 1.34)	0.03	0.0238	87196339	ECM of skeletal muscle
Endoglin (*ENG*)	430	0.89 (0.42, 1.36)	0.03	0.0238	4557555	Regulation of ECM synthesis
Pigment epithelium-derived factor (*PEDF*)	499	1.23 (0.57, 1.88)	0.03	0.0238	39725934	Anti-angiogenesis
Anthrax toxin receptor 1 (*ANTXR1*)	338	0.80 (0.37, 1.23)	0.04	0.0238	14149904	ECM homeostasis
Fibroblast activation protein (*FAP*)	422	0.77 (0.35, 1.19)	0.03	0.0239	16933540	ECM remodeling
Chemokine (C-C motif) ligand 14 (*CCL14*)	77	1.66 (0.7, 2.61)	0.17	0.0369	14589961	Intracellular Ca^2+^ regulation
Complement factor D (*CFD*)	499	1.24 (0.52, 1.96)	0.02	0.0404	42544239	Complement activation or insulin regulation
IGF I (*IGF-I*)	173	0.66 (0.27, 1.06)	0.06	0.046	163659901	Growth factor
Threonine synthase–like 1 (*THNSL1*)	166	0.96 (0.38, 1.54)	0.06	0.046	153792148	Amino acid biosynthesis
Cartilage oligomeric matrix protein (*COMP*)	499	0.65 (0.26, 1.04)	0.02	0.046	40217843	ECM (noncollagenous glycoprotein)
Perlecan (*HSPG2*)	492	1.26 (0.5, 2.02)	0.02	0.046	126012571	ECM (proteoglycan)
Carnosinase 1 (*CNDP1*)	499	0.39 (0.15, 0.62)	0.02	0.046	21071039	Carnosine hydrolase
Negative associations						
Heparin cofactor II (*HCF2*)	499	−0.99 (−1.53, −0.46)	0.03	0.0238	73858566	Thrombin inhibitor
Phosphogluconate dehydrogenase (*PGD*)	97	−1.05 (−1.66, −0.44)	0.28	0.0369	40068518	Enzyme in the pentose phosphate pathway

1 Seventeen proteins quantified by MS and estimated by linear mixed-effects modeling in >10% of the samples that were positively and negatively associated with AMA (*q* < 0.05), listed by the direction and strength of association (in increasing order of *q*). AMA, arm muscle area; ECM, extracellular matrix; IGF, insulin-like growth factor.

2 Number of child plasma samples in which each protein was detected and quantified by MS. One outlier of AMA was excluded; thus, the maximum number of children included in the analysis was 499.

3 Estimated change in AMA (in square centimeters) (95% CI) of children per 50% (1.5 times) increase in the relative abundance of protein.

4 Proportion of variability in AMA explained by protein.

5 Multiple hypothesis testing was corrected by using the false discovery rate.

6 GenInfo sequence number as assigned to all protein sequences by the National Center for Biotechnology Information at the National Library of Medicine, NIH (35).

7 Represented or known molecular function or biological process of protein.

### Plasma proteins associated with BMI-for-age.

BMI, reflecting both lean and fat mass, was lower by −1.14 (95% CI: −1.63, −0.66) *z* scores per 50% increase in relative abundance of myosin light-chain kinase (*q* = 0.0021). This protein was detected in only 74 of 500 children, which explained 38% of the variability in BAZ. It is an enzyme localized in smooth muscle cells and involved in muscle contraction (49). No other proteins were associated with BAZ or differentially abundant between children with low BMI (BAZ <−2) and normal BMI (BAZ ≥−2).

### Plasma proteins associated with WAZ and underweight status.

Weight-for-age was higher by 0.21–0.74 of a *z* score and lower by 0.21–0.96 of a *z* score per 50% increase in relative abundance of positively (*n* = 22) and negatively (*n* = 11) correlated proteins, respectively (**Table 3**). IGF-I, IGFALS, and IGFBP3 showed the strongest positive associations (all *q* < 0.0001), followed by carnosinase 1. In addition to proteins commonly associated with HAZ, BAZ, and AMA, neuropilin and noelin, involved in angiogenesis (50) and neural development (51), respectively, were positively associated with WAZ. Proteins with a variety of functions, including thioredoxin, platelet glycoprotein V, adipocyte plasma membrane-associated protein, chromogranin A, and cathepsin D, were inversely associated with WAZ. In a separate analysis, IGF-I, IGFALS, IGFBP3, carnosinase 1, noelin, and 4 other proteins were 3–18% less abundant and adipocyte plasma membrane-associated protein was 7% more abundant in the plasma of underweight children (WAZ <−2) than children with WAZ ≥−2 (**Supplemental Table 5**).

**TABLE 3:** Plasma proteins positively and negatively associated with WAZ in 6- to 8-y-old Nepalese children (*q* < 0.05)^1^

Protein (gene symbol)	*n*^2^	Change in WAZ^3^	*R*^2^ 4	*q*^5^	Accession^6^	Molecular function or biological process^7^
Positive associations						
IGF-binding protein, acid labile subunit (*IGFALS*)	499	0.74 (0.6, 0.89)	0.26	6.59 × 10^−21^	4826772	IGF binding
IGF-binding protein 3 (*IGFBP3*)	499	0.57 (0.43, 0.71)	0.20	3.80 × 10^−13^	62243068	IGF binding
IGF I (*IGF-I*)	173	0.37 (0.23, 0.51)	0.17	5.13 × 10^−05^	163659901	Growth factor
Carnosinase 1 (*CNDP1*)	499	0.21 (0.12, 0.31)	0.11	0.0011	21071039	Carnosine hydrolase
Pigment epithelium-derived factor (*PEDF*)	499	0.58 (0.32, 0.84)	0.11	0.0021	39725934	Anti-angiogenesis
Perlecan (*HSPG2*)	492	0.58 (0.28, 0.89)	0.10	0.0133	126012571	ECM (proteoglycan)
Noelin (*OLFM1*)	464	0.44 (0.21, 0.67)	0.12	0.0142	17136143	Neural development
Afamin (*AFM*)	499	0.36 (0.16, 0.56)	0.10	0.0166	4501987	Vitamin E transport
Attractin (*ATRN*)	499	0.51 (0.23, 0.79)	0.10	0.0166	21450863	Inflammatory response
Anthrax toxin receptor 1 (*ANTXR1*)	338	0.31 (0.14, 0.47)	0.08	0.0166	14149904	ECM homeostasis
Collagen VI, α1 (*COL6A1*)	471	0.32 (0.14, 0.51)	0.11	0.0259	87196339	ECM of skeletal muscle
Neuropilin 1 (*NRP1*)	457	0.31 (0.13, 0.49)	0.13	0.0265	66864913	Angiogenesis
Complement factor D (*CFD*)	499	0.5 (0.21, 0.79)	0.10	0.0265	42544239	Complement activation or adipokine
Endoglin (*ENG*)	430	0.32 (0.13, 0.51)	0.04	0.0290	4557555	Regulation of ECM synthesis
Apolipoprotein L1 (*APOL1*)	499	0.28 (0.12, 0.45)	0.10	0.0290	211938442	Lipid transport
Thrombospondin 4 (*THBS4*)	451	0.24 (0.1, 0.37)	0.08	0.0290	31543806	ECM (noncollagenous glycoprotein)
Chemokine (C-C motif) ligand 14 (*CCL14*)	77	0.65 (0.26, 1.05)	0.13	0.0349	14589961	Intracellular Ca^2+^ regulation
Fibroblast activation protein, α subunit (*FAP*)	422	0.28 (0.11, 0.45)	0.06	0.0354	16933540	ECM remodeling
Cadherin 5 (*CDH5*)	499	0.34 (0.13, 0.55)	0.10	0.0424	166362713	Endothelial cell-cell adhesion
Collagen VI, α3 (*COL6A3*)	471	0.35 (0.13, 0.56)	0.12	0.0424	55743106	ECM of skeletal muscle
Tetranectin (*CLEC3B*)	499	0.43 (0.16, 0.71)	0.10	0.0465	156627579	Bone matrix
Mannan-binding lectin serine protease 2 (*MASP2*)	112	0.67 (0.23, 1.1)	0.24	0.0465	21264363	Complement activation
Negative associations						
Protein S100-A12 (*S100A12*)	376	−0.21 (−0.31, −0.1)	0.06	0.0077	5032059	Immune response
Myosin light-chain kinase, smooth muscle (*MLCK*)	74	−0.96 (−1.44, −0.48)	0.18	0.0077	116008190	Muscle contraction
Adipocyte plasma membrane–associated protein (*APMAP*)	485	−0.28 (−0.43, −0.14)	0.06	0.0133	24308201	Adipocyte differentiation
IGF-binding protein 2 (*IGFBP2*)	499	−0.28 (−0.44, −0.13)	0.10	0.0166	55925576	IGF binding
Chromogranin A (*CHGA*)20	339	−0.27 (−0.43, −0.11)	0.08	0.0354	4502805	Neuroendocrine secretory protein
Thioredoxin (*TXN*)	311	−0.25 (−0.41, −0.1)	0.07	0.0405	50592994	Redox homeostasis
Platelet glycoprotein V (*GP5*)	445	−0.24 (−0.39, −0.09)	0.07	0.0424	4758460	Platelet aggregation
Stonin-2 (*STON2*)	125	−0.34 (−0.55, −0.12)	0.09	0.0445	21361863	Endocytosis
Phosphogluconate dehydrogenase (*PGD*)	97	−0.44 (−0.73, −0.16)	0.17	0.0465	40068518	Enzyme in the pentose phosphate pathway
Cathepsin D (*CTSD*)	307	−0.23 (−0.37, −0.08)	0.13	0.0465	4503143	Lysosomal protease
Hemopexin (*HPX*)	499	−0.48 (−0.79, −0.17)	0.10	0.0491	11321561	Heme transport

1 Thirty-three proteins quantified by MS and estimated by linear mixed-effects modeling in >10% of the samples that were positively and negatively associated with WAZ (*q* < 0.05), listed by the direction and strength of association (in increasing order of *q*). ECM, extracellular matrix; IGF, insulin-like growth factor; WAZ, weight-for-age *z* score.

2 Number of child plasma samples in which each protein was detected and quantified by MS. One outlier of WAZ was excluded; thus, the maximum number of children included in the analysis was 499.

3 Estimated change in WAZ (95% CI) of children per 50% (1.5 times) increase in the relative abundance of protein.

4 Proportion of variability in WAZ explained by protein.

5 Multiple hypothesis testing was corrected by using false discovery rate.

6 GenInfo sequence number as assigned to all protein sequences by the National Center for Biotechnology Information at the National Library of Medicine, NIH (35).

7 Represented or known molecular function or biological process of protein.

A total of 38 plasma proteins were positively or negatively associated with ≥1 evaluated anthropometric measurement or index. A full correlation matrix of all proteins is provided in **Supplemental Figure 2**.

## Discussion

In this typical, rural-plains region of Nepal, chronic undernutrition manifests as short stature and thinness in the early school-aged years. We explored the plasma proteome of children in this area to discover proteins associated with anthropometric measures of skeletal bone and muscular growth and subcutaneous fatness. Our results revealed that certain plasma proteins were associated with HAZ, WAZ, BAZ, and upper AMA, although no associations were observed between plasma protein abundance and indicators of subcutaneous fat (i.e., AFA and triceps and subscapular skinfolds) below a false discovery threshold of 5%. These results collectively suggest that the plasma proteome may reflect underlying biological processes of regulating lean tissue growth in this population of undernourished children.

Strong positive associations between IGF-I and its 2 binding proteins (IGFALS and IGFBP3) and height-for-age, weight-for-age, and AMA support the known anabolic effects of IGF-I axis on skeletal muscle and bone tissue growth (52, 53). IGF-I is known to be responsive to both acute and chronic nutritional status (54). This is consistent with our observation that IGF-I and the 2 binding proteins were associated with skeletal muscle mass and the long-term process of linear bone growth (5). Interestingly, the 2 IGF-I–binding proteins showed stronger associations than IGF-I. Unbound IGF-I can only briefly remain in the bloodstream before forming a ternary complex with its circulating binding proteins to extend its half-life (43). Thus, we postulate that abundance of the 2 IGF-I–binding proteins may be more sensitive to long-term nutritional status than plasma abundance of IGF-I in this chronically undernourished child population.

To a lesser extent than the IGF-I ternary complex, carnosinase 1 was also positively associated with attained height, weight, and musculature. It is an enzyme that hydrolyzes the dipeptide carnosine into its constituent amino acids, β-alanine and histidine (39). Carnosine is an antioxidant that is abundantly found in skeletal muscle and rapidly hydrolyzed in the plasma. Although little is known about the role of carnosinase 1 in bone growth, studies have noted its reduced activity or a low concentration in alcoholic patients with metabolic myopathy (55), anorexic patients with severe protein-energy deficiency (56), and cancer patients with cachexia (57). Our finding supports the theory that plasma abundance of this protein may be expected to positively correlate with muscle mass and, thus, reflect adequacy of lean tissue. Because skeletal muscle is essential to support mechanical functions of the skeleton (58), and height-for-age and AMA were positively correlated in these study children (*r* = 0.46), carnosinase 1 may be a candidate biomarker for interdependent musculoskeletal growth and function in growing children.

Several proteins involved in the immune response, nutrient metabolism, and bone remodeling were specific in their associations with height-for-age. S100A12 is predominantly secreted from activated neutrophils and is considered a sensitive marker of an innate inflammatory response (42). It is not clear which mechanisms may underlie the inverse association between S100A12 and attained stature. Elevated plasma or fecal S100A12 has been found associated with chronic intestinal inflammation in children (59, 60). Thus, one plausible explanation is that it may reflect subclinically impaired gut function, which is a risk factor for child growth faltering in developing countries (61). Another correlate of height-for-age was afamin, a protein in the albumin family with emerging pleiotropic roles in glucose, lipid, and bone metabolism beyond its role and associations with vitamin E transport (17, 37). Finally, tetranectin, also solely associated with stature, is an abundant protein in bone cartilage that plays a role in bone mineralization during osteogenesis (40). In summary, proteins associated with HAZ are involved in a variety of biological functions, and their gradual differences in plasma abundance by attained height (Figure 2) suggest combined effects of multiple pathways on skeletal development in children.

Skeletal musculature of studied children, indicated by AMA, covaried with relative abundance of proteins of extracellular matrix origin, including its major structural components (collagen and proteoglycan) and homeostasis-regulating proteins. The extracellular matrix is a structural framework of connective tissue that is abundant in skeletal muscle (7). Its constant turnover occurs through interactions with myocytes that include cell-extracellular matrix adhesion, growth factor and membrane receptor signaling, and matrix proteolysis by proteinases (7, 62). These interactions are critical for muscle cell attachment, migration, proliferation, and survival (7). The observed moderate, positive correlations between extracellular matrix–related proteins (Figure 3) are likely to reflect a network of dynamics involving the extracelluar matrix that promotes skeletal muscle development.

One protein, myosin light-chain kinase (MLCK), was negatively associated with age-adjusted BMI. Although BMI does not distinguish lean from fat mass, a substantial literature supports localization of MLCK in muscle tissue where it regulates muscle contraction (49). Studies have reported an *MLCK* genotype to be associated with exertional muscle damage and subsequent leakage of intracellular proteins into circulation in response to high physical demand (63, 64). Thus, it is possible that children exposed to chronic stresses of malnutrition or low-grade inflammation (15) experience greater systemic breakdown of muscle tissue resulting in a higher abundance this protein in plasma (65).

Noteworthy in this study was a virtual lack of association between detectable plasma proteins and measures of adiposity. One possible explanation is that skinfolds and BMI may become less valid indicators of body fat in pediatric populations with very low adipose tissue (66), such as observed in this study in which average triceps and upper AFA measurements were <10th percentile of American children (32–34). It is also possible that fat mass–regulating or associated proteins in this lean and low-fat child phenotype were too low in plasma abundance to be detected by MS.

As a composite measurement, weight-for-age was associated with the largest and most comprehensive proteome. Because musculoskeletal mass substantially contributes to body weight, the proteome of weight included most proteins that were associated with height and arm muscle mass. In addition, positive correlates included noelin, a protein involved in nervous system development (67). Negative correlates also included proteins expressed during cellular responses to catabolic stress, such as those associated with oxidative damage (thioredoxin) (68) and vascular injury (platelet glycoprotein V). Another negative correlate, cathepsin D, is a lysosomal endopeptidase that can promote tissue degradation (69). Although body weight is nonspecific to body dimension and composition, the proteins identified in this study deserve further exploration because they may represent nutritionally regulated systems or aspects of ponderal growth that cannot be assessed by anthropometry.

Among studies that have applied omics approaches to elucidate how undernutrition may affect childhood growth and associated health outcomes (70–72), this is the first proteomics study to our knowledge that has identified protein biomarkers correlated with multiple facets of attained child growth based on extensive anthropometric data. Because study participants were sampled by a random process from a large population cohort of children residing in an area typical of the terai of Nepal and greater Gangetic flood plains region, we surmise that our findings relating the plasma proteome to child phenotype may be generalizable to similarly undernourished child populations in the region.

A limitation of this cross-sectional analysis is that we cannot assume causal inferences about relations observed between plasma proteins and anthropometric phenotype. More accurate body composition measures, such as provided by bioelectrical impedance analysis or other advanced methods, were not available in this study, which would have allowed us to explore associations between the plasma proteome and visceral or total-body fat stores. Finally, proteins detected by MS were measured on a relative scale. Because the proteomics approach has consistently provided valid direction and strength of association between detected proteins and other nutritional outcomes (14, 16, 17), the identified proteins in this study deserve further validation and replication in other populations to test plasma proteins as nutrition-sensitive biomarkers for clinical and public health practice.

This plasma proteomics study identified known growth-promoting factors and novel plasma proteins associated with anthropometric indicators in rural South Asian children. In particular, proteins were most strongly associated with lean tissue depots and may reflect pathways of lean tissue growth in these children. Because child anthropometric features are associated with immunologic and neurological functions and long-term metabolic health, proteins identified in this study may prove useful in the future as functional biomarkers of nutritional status, growth processes, and population health status.

## Footnotes

10 Abbreviations used: AFA, arm fat area; AMA, arm muscle area; BAZ, BMI-for-age *z* score; HAZ, height-for-age *z* score; IGF, insulin-like growth factor; IGFALS, insulin-like growth factor–binding protein, acid labile subunit; IGFBP3, insulin-like growth factor–binding protein 3; iTRAQ, isobaric Tags for Relative and Absolute Quantitation; LME, linear mixed-effects; MLCK, myosin light-chain kinase; MUAC, midupper arm circumference; S100A12, protein S100-A12; WAZ, weight-for-age *z* score.
